# Idiosyncratic responses of evergreen broad-leaved forest constituents in China to the late Quaternary climate changes

**DOI:** 10.1038/srep31044

**Published:** 2016-08-18

**Authors:** Dengmei Fan, Wan Hu, Bo Li, Ashley B. Morris, Min Zheng, Douglas E. Soltis, Pamela S. Soltis, Zhiyong Zhang

**Affiliations:** 1 Laboratory of Subtropical Biodiversity, Jiangxi Agricultural University, Nanchang, Jiangxi, 330045, China; 2 Department of Biology, Middle Tennessee State University, Murfreesboro, TN 37132, Tennessee, USA; 3 Department of Biology, University of Florida, Gainesville, FL 17 32611, USA; 4 Florida Museum of Natural History, University of Florida, Gainesville, FL 17 32611, USA

## Abstract

Subtropical evergreen broad-leaved forest (EBLF) is one of the most important vegetation types in China. Inferences from palaeo-biome reconstruction (PBR) and phylogeography regarding range shift history of EBLF during the late Quaternary are controversial and should be reconciled. We compared phylogeographic patterns of three EBLF constituents in China, *Castanopsis tibetana*, *Machilus thunbergii* and *Schima superba*. Contrary to a chorus of previous phylogeographic studies and the results of species distribution modelling (SDM) of this study (*in situ* survival during the LGM), the three species displayed three different phylogeographic patterns that conform to either an *in situ* survival model or an expansion-contraction model. These results are partially congruent with the inference of PBR that EBLF was absent to the north of 24° N at the LGM. This study suggests that the constituents of EBLF could have responded idiosyncratically to climate changes during the Late Quaternary. The community assemblages of EBLF could have been changing over time, resulting in no palaeo-analogs to modern-day EBLF, which may be the main reason responsible for the failure of PBR to detect the occurrence of EBLF north of 24° N at the LGM.

Subtropical evergreen broad-leaved forest (EBLF) occurs from 24 to 32° N latitude and 99 to 123° E longitude and covers approximately 25% of China (Fig. 1a), making it one of the most important vegetation types in China^1^,^2^. This biome is heavily influenced by the East Asian monsoon and human expansion and is predicted to be vulnerable to future climate change^3^. However, the potential impacts of future climate change on the ecosystem are poorly quantified because of sparse long-term *in situ* measurements^3^ and the inherent difficulties in predictions of climate change scenarios on biodiversity^4^. Retrospective analyses based on fossil and genetic data have greatly advanced our understanding of species diversity and distribution in response to past climatic changes^5^, thus providing a framework for explaining biotic responses to anthropogenic climate change in the future^6^. However, the range shift of EBLF in response to the late Quaternary climate changes remains a subject of dispute, which may compromise the ability to make informed decisions regarding biodiversity conservation and natural resource management in the EBLF region in response to future climate change.

Over the past 3 million years, the climate on Earth has undergone periodic fluctuations, resulting in complex changes of vegetation distributions^7^. During the last glacial maximum (LGM), for example, the mean annual temperature of the EBLF region was cooler by c. 4–6 °C and drier by c. 400–600 mm/yr according to pollen analyses^8^. Palaeo-biome reconstructions (PBR) based on fossil pollen suggested that at the time of the LGM, the EBLF in subtropical China extended as far south as c. 24°N, a shift of ca. 1000 km relative to today^9^,^10^,^11^. These studies also indicated that EBLF subsequently expanded northward during the Holocene, covering a more extensive range than today, ca. 200 km north of the modern limit (glacial contraction and post-glacial expansion, EI model). In contrast, most previous phylogeographic studies, sometimes combined with species distribution modelling (SDM), have consistently concluded that the component species of EBLF survived the LGM *in situ* in multiple northern refugia and did not experience extensive northward spread from more southern refugia during the Holocene (*in situ* survival model, ISS model) (Supplementary Table S1).

Why are the results from PBR and phylogeography (sometimes coupled with SDM) so different ? On the one hand, the fossil pollen record in central east China (including the EBLF region) is so sparse that unambiguous reconstruction of biome boundaries in this region is difficult^11^. Furthermore, fossil pollen records are always limited in taxonomic resolution^5^, such that the reconstruction of entire biomes based on these data cannot produce a detailed picture of palaeodistributions of EBLF constituents^12^. On the other hand, previous phylogeographic studies of elements of the EBLF are heavily biased towards Fagaceae (e.g., *Castanopsis eyrei*^13^, *Castanopsis fargesii*^14^ and *Quercus glauca*^15^, Supplementary Table S1), and the conclusions may be phylogenetically biased and prone to phylogenetic niche conservatism (the tendency of species to retain ancestral ecological attributes)^16^,^17^. The remaining phylogeographic studies mostly involved species that are ecologically minor components of EBLF (e.g., *Eurycorymbus cavaleriei*^18^, *Rhododendron simsii*^19^ and *Tetrastigma hemsleyanum*^20^, Supplementary Table S1), and the significance of these results may therefore not be representative of the biome as a whole. Importantly, the investigated plants represent merely a small proportion of the total plant biodiversity in the EBLF region (up to 18,000 seed plant species^21^), and the conclusions of previous phylogeographic studies regarding the biome as a whole may therefore be premature. Additional phylogeographic surveys, especially rigorous comparisons among co-distributed dominant species within EBLF (i.e., comparative phylogeography), may provide insights into the divergent results offered by PBR, phylogeographic analyses, and SDM.

Comparative phylogeography (CP) seeks to understand how the geographic ranges of co-distributed species have changed over time^22^. By comparing phylogeographic patterns across species, it may be possible to discern whether changes in distribution and population size represent the influence of extrinsic factors that affected whole communities, or whether they can be ascribed to stochastic variation and other chance factors particular to each organism^23^. Some comparative studies have succeeded in identifying patterns common across many groups of organisms^24^,^25^,^26^. However, idiosyncratic phylogeographic patterns among co-distributed species are commonly reported^27^,^28^,^29^, suggesting that community composition is highly dynamic^29^. If the latter model applies to EBLF in subtropical China, it is conceivable that the absence of EBLF in subtropical China at the LGM might have been caused by alterations in community composition and the changes in relative abundance of EBLF components, rather than by the complete retreat to the south, thus settling the dispute between PBR and phylogeography. However, such a hypothesis has never been proposed and explicitly tested by phylogeographic researchers in China, possibly due to the difficulty of gathering large data sets from multiple co-distributed species.

To reconcile the two scenarios suggested by PBR and phylogeography, this study used the CP approach to test for congruent versus idiosyncratic phylogeographic patterns of multiple species from EBLF in subtropical China. The EBLF zone within China is divided into two subregions (Fig. 1a): the moist EBLF in eastern China influenced by the Pacific monsoon and the semi-moist EBLF in the west determined by the Indian monsoon^2^. Because the climate in the two subregions is quite different (e.g., the annual rainfall is 1000–2000 mm in the moist east and 900–1200 mm in the semi-moist west^1^), very few dominant tree species are shared across the two subregions. Therefore, we only focused on three tree species in the eastern subregion of EBLF, i.e., *Castanopsis tibetana* Hance (Fagaceae), *Machilus thunbergii* Sieb. et Zucc. (Lauraceae), and *Schima superba* Gardn. et Champ. (Theaceae) (Supplementary Table S2; Fig. 1). These species satisfy three criteria: 1) they are all dominant broad-leaved tree species in EBLF and thus play a fundamental role in the ecological functions of EBLF; 2) they belong to different families and genera that avoid the influence of conserved niche within lineages (and thus similar responses to climate change); and 3) they are components of the same forest communities. We surveyed chloroplast DNA (cpDNA) sequence variation in these three tree species and determined their phylogeographic and historical demographic patterns. We also used SDM to hindcast the geographical distribution of the three tree species at the time of the LGM. By providing quantitative and high-resolution predictions of individual palaeodistributions, this method can complement fossil and genetic evidence concerning the past distribution of species and forest communities^30^. By using molecular and SDM approaches, we address the following issues: i) whether the focal species conform to the ISS or the EI model during the late Quaternary; ii) whether they display congruent phylogeographic patterns and demographic histories across a shared landscape; and iii) whether CP is able to reconcile the dispute between PBR and previous phylogeographic studies.

## Results

### Sequence characteristics and genetic diversity

The combined alignments of the two chloroplast regions were 1826 bp (*rpl32–trnL*^(UAG)^ 1313 bp + *psbA–trnH* 513 bp), 1450 bp (1048 bp + 402 bp), and 1399 bp (976 bp + 423 bp), comprising 29 (*rpl32–trnL*^(UAG)^ 17 + *psbA–trnH* 12), 13 (7 + 6), and 13 (8 + 5) polymorphic sites and defining 19, 13, and 9 haplotypes in *C. tibetana*, *M. thunbergii*, and *S. superba*, respectively (Supplementary Table S3). All haplotype sequences are deposited in GenBank (accession numbers: KP892691–KP892749).

Estimates of haplotype diversity (*h*) and nucleotide diversity (π) for each population of each of the three species are summarized in Supplementary Table S2. The overall haplotype diversity was equivalent in *C. tibetana* and *S. superba* (*h*_T_ = 0.797 and 0.765, respectively), but higher than that in *M. thunbergii* (*h*_T_ = 0.644). *Machilus thunbergii* displays the lowest nucleotide diversity, while *C. tibetana* has the highest. The average population genetic diversity values are all considerably low in *C. tibetana*, *M. thunbergii*, and *S. superba* (*h*_S_ = 0.151, 0.165, and 0.136, respectively).

### Phylogeographic structure

**Figure 1:**
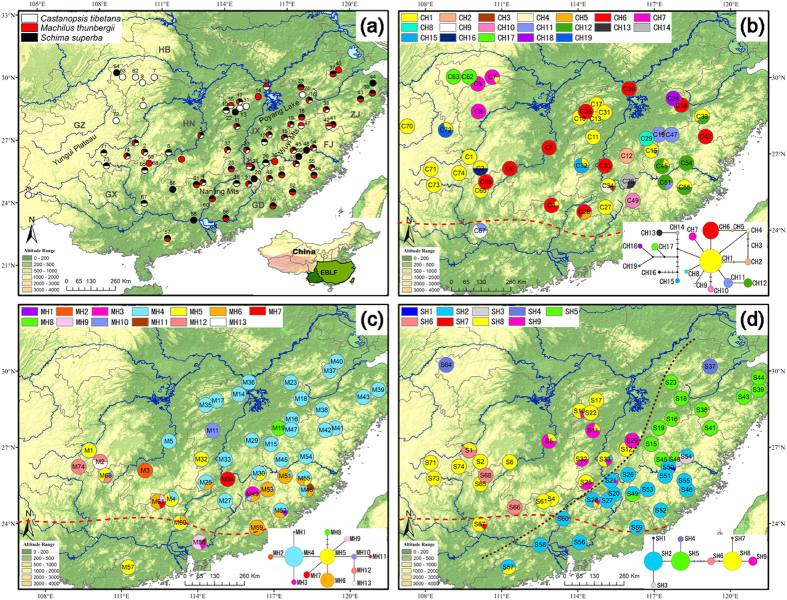
(**a**) Locations of sampled populations of *Castanopsis tibetana*, *Machilus thunbergii*, and *Schima superba*. The circle in the inset illustrates the distribution range of subtropical evergreen broad-leaved forest (EBLF) in China and the dashed line shows the boundary between two subregions of EBLF. (**b**) Geographic distribution and median-joining network of *C. tibetana* haplotypes (CH1–19). (**c**) Geographic distribution and median-joining network of *M. thunbergii* haplotypes (MH1–13). (**d**) Geographic distribution and median-joining network of *S. superba* haplotypes (SH1–9). Brown dashed lines denote lineage divergences identified by network and SAMOVA analysis. For each network in (**b**–**d**), the size of circles corresponds to the frequency of each haplotype. Vertical bars indicate unsampled or extinct haplotypes. Each solid line represents one mutational step that interconnects two haplotypes. Each population code in Supplementary Table S2 is preffixed by a letter C in (**b**), M in (**c**) and S in (**d**) that particularly denotes the sampled populations of *Castanopsis tibetana*, *Machilus thunbergii* and *Schima superba*, respectively. One population (M69) remote from others in *Machilus thunbergii* is not shown in (**c**). The red dashed lines show the approximate northern borders of EBLF during the LGM based on PBR (modified after Harrison *et al*., 2001). Maps were generated using ArcGIS version 9.3 (http://www.esri.com/software/arcgis/arcgis-for-desktop) and Adobe Illustrator CS3 13.0 and modified using Adobe Photoshop CS 8.0.

In *C. tibetana*, samova failed to uncover any reliable population genetic grouping, and there was no apparent structure in the haplotype network (Fig. 1b). Except for two relatively widespread haplotypes, CH1 and CH6, which were found in 22 (49%) and 11 (24%) populations, respectively, most haplotypes were confined to a specific region. Fifteen polymorphic populations and 13 haplotypes (CH2–5, CH8–10, CH13–16 and CH18–19) private to a single population were scattered across the species’ range.

There was also no apparent association between haplotypes and geography according to the samova analysis in *M. thunbergii*. However, the haplotype distribution in *M. thunbergii* was distinct from that in *C. tibetana* (Fig. 1c). The most common haplotype, MH4, occurred in 32 (70%) populations and was fixed in 22 of them, especially those at higher latitudes ( ≥ 26°N). The second and third most common haplotypes, MH5 and MH6, were shared among 9 (20%) and 10 (22%) populations, respectively. Of the 10 rare haplotypes remaining, five (MH1, MH2, MH8, MH10, and MH13) were found in a single population (M52, M3, M19, M11, and M2, respectively), and five (MH3, MH7, MH9, MH11, and MH12) were shared by two or three populations. Most haplotypes (except for MH4, MH8, and MH10) and 14 polymorphic populations occurred at lower latitudes ( ≤ 26°N).

In the samova analysis of *S. superba*, the value of *F*_CT_ reached a plateau at 0.894 when the number of groups was two (*K* = 2). As shown in Fig. 1d, the two groups largely correspond to western and eastern parts of the distribution range. The western group was dominated by SH8. The eastern group had two subgroups, the southern one nearly fixed for SH2 and the northern one for SH5. The haplotype network recovered all western haplotypes (SH6–9) as a clade, which was separated by five mutational steps from an eastern clade (containing SH1–5). Several populations in the contact area of the two groups showed a mixture of two clades (S21, S28, S33, and S57).

**Figure 2:**
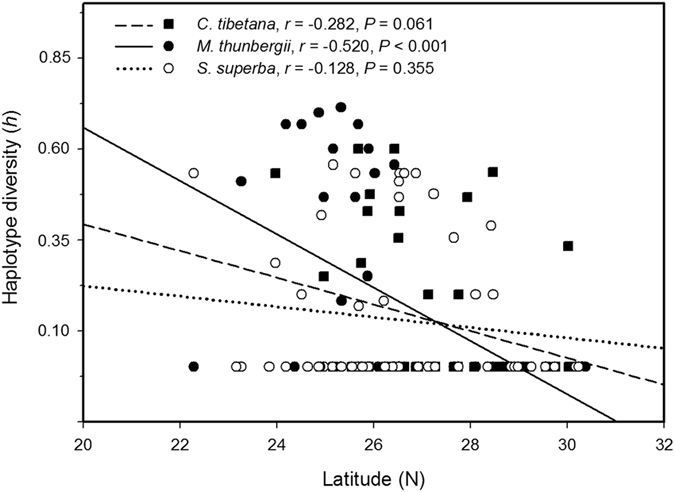
The relationship between haplotype diversity (*h*) and latitude (°N) in three broad-leaved evergreen tree species in subtropical China.

In the multiple regression analyses of genetic variation, haplotype diversity was correlated negatively with latitude in *M. thunbergii* (*P* < 0.001), but not in *C. tibetana* or *S. superba* (Fig. 2). Nonetheless, a *U*-test showed that *N*_ST_ was significantly larger than *G*_ST_ in *S. superba* (*N*_ST_ = 0.906; *G*_ST_ = 0.825; *U* = 2.81, *P* < 0.01), indicating phylogeographic structure of haplotype distribution, but this was not significant in either *C. tibetana* (*N*_ST_ = 0.851; *G*_ST_ = 0.815; *U* = 0.90, *P* > 0.05) or *M. thunbergii* (*N*_ST_ = 0.767; *G*_ST_ = 0.752; *U* = 0.18, *P* > 0.05).

### Historical demography

**Figure 3:**
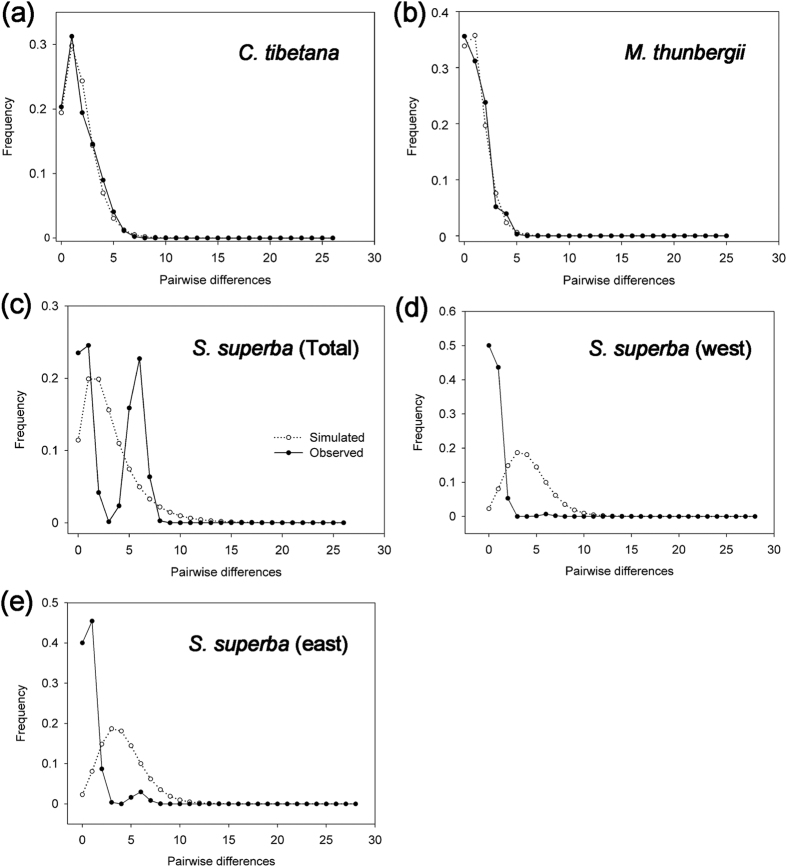
Distribution of the number of pairwise nucleotide differences for cpDNA sequence data in (**a**) *Castanopsis tibetana* as a whole, (**b**) *Machilus thunbergii* as a whole, (**c**) *Schima superba* as a whole, (**d**) eastern populations of *Schima superba* and (**e**) western populations of *Schima superba*. The solid line shows observed distributions of differences among haplotypes whereas the dashed line represents simulated distributions under a model of sudden (stepwise) population expansion (Rogers & Harpending, 1992).

For *C. tibetana*, the neutrality tests showed that both Tajima’s *D* (*D* = −1.011, *P* = 0.157) and *F*_S_ (*F*_S_ = −2.888, *P* = 0.026) were negative. The mismatch distribution for haplotypes was unimodal (Fig. 3a) that was statistically consistent with the expansion model (*P* values > 0.05 for *SSD* and *H*_*Rag*_; Table 1). Despite a slight recent decrease in population size detected by our BSP analysis (Supplementary Fig. S1a), the data suggest [we prefer] that *C. tibetana* likely experienced past expansion. Based on the corresponding *τ* value, the demographic expansion event was estimated to have occurred at 9,600 (95% highest posterior density, HPD: 0–53,000) years bp, although this estimate should be treated with caution given the wide confidence interval (Table 1).

For *M. thunbergii*, the negative Tajima’s *D* (*D* = −0.746, *P* = 0.252) and *F*_S_ (*F*_S_ = −2.467, *P* = 0.041), unimodal mismatch distribution and positive values of *SSD* and *H*_*Rag*_ (*P* > 0.05) consistently suggested a past expansion event (Table 1, Fig. 3b). The expansion event was estimated to have occurred approximately 3,700 (95% HPD: 0–33,000) years bp based on the *τ* value. Our BSP analysis also showed that *M. thunbergii* populations experienced a recent sharp increase in population size at *c*. 4 ka (Supplementary Fig. S1b).

For *S. superba*, the neutrality tests gave contrasting results, with a non-significant positive Tajima’s *D* (*D* = 1.93, *P* = 0.979) and a significant positive *F*_S_ (*F*_S_ = 4.656, *P* = 0.013). Despite a good statistical fit of the expansion model (*P* values > 0.05 for *SSD* and *H*_*Rag*_; Table 1), the mismatch distribution for *S. superba* was bimodal, which may reflect the regional population structure of the species due to the west-east divergence (Fig. 3c). In support of this explanation, when analyzed separately, the western populations showed a unimodal distribution (Fig. 3b) that was statistically consistent with the expansion model (*P* values > 0.05 for *SSD* and *H*_*Rag*_; Table 1). This expansion event was dated at 6,100 (95% HPD: 0–115,100). When examined separately, the eastern populations showed a bimodal distribution (Fig. 3c), because this group was further structured into two subgroups (Fig. 3c). A BSP analysis detected a slight population increase in both the western and eastern groups (Supplementary Fig. S1c, S1d). Taken together, the data suggest that *S. superba* may have experienced regional post-glacial expansion from localized refugia.

### Species distribution modeling

**Figure 4:**
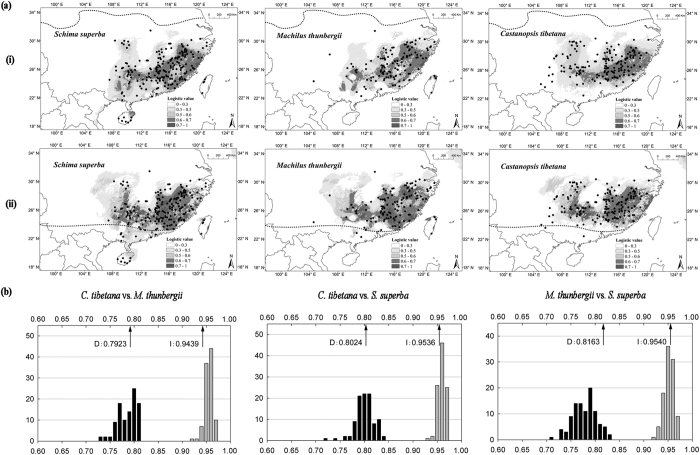
(**a**) Predicted distributions of *Castanopsis tibetana*, *Machilus thunbergii* and *Schima superba* based on species distribution modeling (i) at present (1950–2000), (ii) at the Last Glacial Maximum (LGM; *c*. 21 kya). Species distribution models were established with bioclimatic variables on the basis of extant occurrence points (black dots) of the three species using maxent version 3.3.3 k (http://www.cs.princeton.edu/~schapire/maxent/). The dashed lines indicate the northern borders of EBLF today and during the LGM inferred from PBR (modified after Harrison *et al*., 2001). Maps were generated using ArcGIS version 9.3 (http://www.esri.com/software/arcgis/arcgis-for-desktop). (**b**) The results of identity tests using enmtools version 1.3. The black Bar indicates the null distributions of *D*, and gray bar indicates the null distributions of *I*. Both are generated from 100 randomizations. *X*-axis indicates value of *I* and *D*, *Y*-axis indicates number of randomizations. The arrow indicates the value in actual maxent runs. The Histograms were drawn using SigmaPlot 10.0.

For each species at present and LGM, the areas under the ‘Receiver Operating Characteristic (ROC) Curve’ (AUC) all have values > 0.95, indicating good predictive model performance. In general, the predicted distributions of the three species under present conditions are similar to their actual distributions in mainland China, each with a potentially patchy range in mountainous areas of subtropical China and a slightly more continuous range for *C. tibetana*. At the LGM, the potential ranges of the three species contracted a little to the south in the eastern part of their ranges. However, most of the current occurrences are assigned high suitability at the LGM, suggesting overall range stability (Fig. 4a). Therefore, the three species did not retreat entirely to the tropical south during the LGM. SDM analyses found that the niches for the three species were very similar (Fig. 4b). ENMTOOLS showed that empirically observed values for I and D were comparable to those expected from pseudoreplicated data sets in all paired analyses (*C. tibetana* vs. *M. thunbergii*, *C. tibetana* vs. *S. superba* and *M. thunbergii* vs. *S. superba*) (Fig. 4b).

## Discussion

### Idiosyncratic responses of three dominant trees in EBLF to the late Quaternary climate changes

Recently, phylogeographic patterns of subtropical EBLF constituents in China have received increasing attention (Supplementary Table S1). These studies consistently revealed limited or regional post/inter-glacial expansion from northern localized refugia (ISS model), rather than extensive northward spread from more southerly located refugia (EI model) as inferred from palaeo-biome reconstruction (PBR) based on the fossil pollen record^9^,^10^,^11^. However, we found that three dominant tree species in EBLF, *C. tibetana*, *M. thunbergii* and *S. superba*, have different range shift trajectories during the late Quaternary, which cannot be predicted purely from either the ISS or EI model.

*Castanopsis tibetana* (Fagaceae), displays a pattern of multiple northern refugia (private haplotypes and polymorphic populations occurring in the north, Fig. 1b) and regional postglacial expansion, which is consistent with the ISS model. It exhibits the highest genetic diversity among the three focal species, suggesting that more glacial refugia could have maintained a larger historical population size. All members of Fagaceae in EBLF examined so far show similar phylogeographic patterns (i.e., *Castanopsis eyrei*^13^, *Castanopsis fargesii*^14^ and *Quercus glauca*^15^). The phylogeographic congruence among members of Fagaceae may be explained by shared population histories because all these species are common in EBLF and always constitute the canopy of the forest^31^. However, an alternative explanation for the congruence may be niche conservatism among closely related species^17^. Based on a meta-analysis of phylogenies from many studies in the Southern Hemisphere, Crisp *et al*.^32^ concluded that closely related species are more ecologically similar than would be expected by chance. Therefore, similar ecological requirements due to phylogenetic relatedness among members of Fagaceae in EBLF may account for their phylogeographic congruence, at least in part.

In contrast to *C. tibetana*, *Machilus thunbergii* has the lowest genetic diversity of the three focal species and shows a significant negative correlation between genetic diversity and latitude (Fig. 2). The results suggest that it conforms to a typical pattern of pioneer or leading-edge expansion, which is expected by the classical EI model^33^. This is the first study that reports the EI model for constituents of EBLF in subtropical mainland China, although evidence of extensive range expansion has been implicated in two case studies of Taiwanese plants. Wu *et al*.^34^ found that *Machilus kusanoi* in Taiwan (most of which is covered by EBLF, Fig. 1a) experienced strong post-glacial range expansion. *Machilus thunbergii* was also investigated in Wu *et al*.^34^: range expansion is also evident in this species because of low genetic diversity among populations and the presence of a widespread haplotype, similar to the pattern observed in mainland China in our study. For another member of Lauraceae, *Cinnamomum kanehirae*, Liao *et al*.^35^ also detected significant spatial range expansion in Taiwan. Together, these studies suggest that some constituents of EBLF in subtropical China (e.g., several species of Lauraceae), may have retreated to the south during the LGM and recolonized the present EBLF region during the Holocene. This conclusion is congruent with recent findings of warm-temperate deciduous forest species in subtropical China. While most species of this type of vegetation exhibit a pattern of multiple refugia and limited range expansion (reviewed by Liu *et al*.^36^), some species show evidence of extensive range expansion^37^,^38^.

All populations of *Schima superba* were divided into two groups by the samova analysis (Fig. 1d). Furthermore, the eastern group can be subdivided into two subgroups. This pattern is an expected consequence of long-term isolation among multiple refugia and subsequent localized range expansion. However, compared to *C. tibetana*, *S. superba* shows significant phylogeographic structure because the major groups correspond to two highly differentiated clades (Fig. 1d). The east-west divergence in eastern subtropical China has also been observed in *Rhododendron simsii*^19^ and *Tsuga*^39^ and may reflect the strong landscape effects of the Wuyi Mountains and the Poyang Lake valley as natural barriers to dispersal. However, such barriers may be semi-permeable and not applicable to all species, because similar divergence has not been observed in *C. tibetana* and *M. thunbergii*, which occupy the same region.

Taken together, in contrast to phylogeographic congruence of EBLF constituents in subtropical China in previous reports^13^,^14^,^15^, this study revealed diverse phylogeographic patterns in three dominant trees, i.e., multiple refugia (across the range) without significant phylogeographic structure in *C. tibetana*, glacial retreat and post-glacial recolonization in *M. thunbergii*, and multiple refugia with significant phylogeographic structure in *S. superba*. This observation agrees with the suggestion of many CP studies that congruent phylogeographic patterns are not necessarily the rule for co-distributed species because of idiosyncrasies in their biological and ecological characteristics, short duration of sharing geographical distribution, and/or different evolutionary histories of genes in the species studied (reviewed by Gutiérrez-García & Vázquez-Domínguez^40^). The results of this study also support the view that communities are not cohesive units that may be broken up and reformed in different configurations repeatedly and regularly on time scales of a few thousand years^41^.

### Reconciling palaeo-biome reconstruction and phylogeography

Although there are contradictory results in a few cases^42^,^43^, the fossil record and phylogeography are largely synergistic in the inferences of past distribution of organisms^5^. Therefore, the distinct scenarios of the past distribution of EBLF in subtropical China inferred from PBR and phylogeography are quite unusual and need to be reconciled. In this study, we found that the phylogeographic patterns of *C. tibetana* and *S. superba* are congruent with the results of previous phylogeographic studies of EBLF constituents. However, *M. thunbergii* displays a typical pioneer or leading-edge expansion pattern, which conforms to the inference of PBR^9^,^10^,^11^, although there is a small difference in the northern limit at the LGM between *M. thunbergii* phylogeography (26°N) and PBR (24°N). These results suggest that the inference of previous phylogeographic studies could be biased by stochastic variance among species and phylogenetic niche conservatism within Fagaceae. More phylogeographic surveys, particularly CP studies with the same sampling strategy and molecular markers, may provide an opportunity to reconcile the discrepancy between PBR and phylogeography.

Despite the fact that some species in EBLF such as *M. thunbergii* retreated to the south during the LGM, the complete absence of EBLF to the north of 24°N at that time suggested by PBR is still contrary to the phylogeographic patterns of most plant species characteristic of this vegetation. Although additional paleopalynological investigations are needed to verify the conclusion of previous PBR, the absence of EBLF to the north of 24°N during the LGM cannot be simply ascribed to the sparse pollen record. In fact, by comparing fossil records of individual forest plants and palaeo-biome maps in North America, Williams *et al*.^41^ also found that the results of PBR sometimes did not match with the original fossil records of individual plants. This observation was attributed to information loss in PBR about the distribution and abundance of individual taxa during the categorization of pollen taxa to one or more plant functional types (PFTs) and biomes^44^ and the violation of the assumption of community stability. According to our CP results, EBLF may have survived in the north during the LGM, but with reduced distribution and abundance (as suggested by the strong evidence for regional postglacial expansion in both *C. tibetana* and *S. superba*) as well as with a different species composition (as suggested by the evidence that *M. thunbergii* retreated to the south). This kind of community dynamics makes it likely that PBR would omit some EBLF components in the fossil record or misidentify the “EBLF” of the LGM as other types of vegetation because the LGM “EBLF” has no counterpart in the present vegetation^41^. This conclusion suggests that CP at the community level is capable of providing a full understanding of historical community dynamics^22^, avoiding the stochastic variance inherent in single-species phylogeographic studies and identifying the artifacts specific to PBR. Therefore, CP offers an insightful perspective for testing competing biogeographic hypotheses evoked by different disciplines.

### The difference between comparative phylogeography and species distribution modeling

Our predicted distributions of three dominant tree species showed that three species survived *in situ* during the LGM (Fig. 4a), consistent with the SDM results of a few EBLF constituents^13^,^14^,^15^. However, these predictions are only partially congruent with the patterns observed in the CP study conducted here, because *M. thunbergii* exhibits an expansion-contraction pattern. As with any model, SDMs rely on many underlying assumptions and thus incorporate uncertainties^45^. One of the most important assumptions in SDMs is niche stability over the study period^30^. However, niche stability may be affected by either changes in biotic interactions in the community or evolutionary adaptation to the biotic and abiotic environment (i.e. changes in realized niches and fundamental niches)^4^. While fundamental niches are less likely to evolve over relatively short periods, such as the LGM to the present, shifts in the realized niche may occur due to changes in biotic interactions^30^. For example, Stewart *et al*.^46^ suggested that extinction of competitors owing to environmental change and small patch size in glacial refugia could change community structure and thus alter a species’ realized niche.

The three species studied here are canopy trees that occupy similar niches (Fig. 4b), implying that competition may exist among them. Such competition could have become much more severe when the EBLF retreated to small glacial refugia because of the low carrying capacity within refugia^47^. It is well known that Fagaceae-dominated vegetation types are climax communities in the EBLF region^48^, indicating that these taxa may have the competitive advantage over other EBLF associates^49^. Therefore, it is reasonable to postulate that *M. thunbergii* might have been a weak competitor that could have been eliminated from glacial refugia by stronger competitors such as *C. tibetana*. Thus, the different results from phylogeography (Fig. 1c) and SDMs (Fig. 4a) for *M. thunbergii* may reflect the fact that it had fewer opportunities to occupy the full extent of its fundamental niche during the LGM because of its poor competitive ability. Future studies using SDMs that account for biotic interactions^50^ might provide projected distributions with higher biological realism that might be better reconciled with phylogeographic inferences.

### The limitations of this study

Comparative phylogeography is a powerful tool for addressing community dynamics in distribution and abundance at the regional scale. However, the conclusions of this study might be subject to two limitations. First, because of financial constraints, this study was limited to two cpDNA markers to allow for a large number of individuals and populations to be surveyed. However, a given spatial genetic structure inferred by cpDNA reflects the history of maternal lineages, as recorded by seed movement, whereas pollen flow, as measured by the nuclear genome, may be more extensive than seed movement, and stochastic processes may lead to a wide range of genetic patterns^27^. Therefore, the patterns observed here should ideally be confirmed using nuclear molecular markers. Second, the three species studied here have a variety of congeners in the EBLF of subtropical China^31^, and hybridization among them is a point of concern for phylogeographic and population genetic studies^51^. However, this situation is difficult to avoid because almost all dominant tree genera in EBLF are species-rich taxa^31^. To limit the potential influence of hybridization on our work, we only included samples from individuals exhibiting typical morphological traits identified by experienced botanists, and we abandoned individuals or populations where morphologically similar species co-occurred. Further investigations on the influence of hybridization by comparing phylogeographic structures of congeneric species (e.g., Saeki *et al*.)^51^ in EBLF may complement the conclusions of this study. Despite these caveats, we believe that the basic conclusions of this study are robust because the phylogeographic pattern of multiple refugia in subtropical China observed in *C. tibetana* and *S. superba* has been repeatedly revealed in the past (see review in Liu *et al*.)^36^. Furthermore, the pattern of glacial retreat and post-glacial recolonization, although rarely reported in the past, has been stressed in a few recent studies^37^,^38^. The main finding of idiosyncratic responses of EBLF constituents in China to the late Quaternary climate changes suggests that the community assemblages of EBLF have changed over time and there is no palaeo-analog to the modern-day EBLF, which explains the discrepancy between PBR and phylogeography.

## Materials and Methods

### Population sampling

Our sampling strategy for phylogeographic analyses was to collect a few individuals (1–13 spaced at least 50 m for each population) from many localities to sufficiently cover the entire geographic range of each species: 332 individuals/45 populations of *C. tibetana*, 392/46 of *M. thunbergii*, and 515/54 of *S. superba*, were sampled (Supplementary Table S2; Fig. 1). All voucher specimens were deposited in the Herbarium of Jiangxi Agricultural University (JXAU).

### DNA extraction, PCR amplification, and sequencing

Total genomic DNA was extracted from *ca.* 20 mg of silica-dried leaf material using a modified cetyltrimethyl ammonium bromide (CTAB) protocol^52^. After preliminary screening of several chloroplast fragments, we chose the *psbA–trnH* and *rpl32–trnL*^(UAG)^ intergenic spacer (IGS) regions for the full phylogeographic survey because they could be successfully amplified in all three species and contained relatively rich polymorphic sites.

PCRs were performed in 20-μl volume containing 50–100 ng genomic DNA, 0.5 μmol/L of each primer, and 10 μl 2 × Taq PCR MasterMix [0.1 U *Taq* polymerase/μl, 0.5 mmol/L dNTP each, 20 mmol/L Tris-HCL (pH 8.3), 100 mmol/L KCl, and 3 mmol/L MgCl_2_; Tiangen]. PCR amplifications were conducted under the following conditions: 5 min at 80 °C, 31–35 cycles of 1 min at 95 °C, 1 min of annealing at 50–58 °C, followed by a ramp rate of 0.3 °C/s to 65 °C, and extension at 65 °C for 4 min, with a final 5-min extension at 65 °C. PCR products were purified using TIANgel Midi Purification Kit (Beijing Tiangen Biotech Co., LTD) prior to sequencing. Sequencing was carried out from both directions using the amplification primers. Sequences containing microsatellites and singletons in some individuals were amplified and sequenced twice to exclude the possibility of PCR errors.

### Phylogeographic and population demography analyses

All sequences for each region were edited with sequencher (GeneCodes Corporation, Ann Arbor, MI, USA) and were aligned using clustal_x 1.81^53^. Indels and inversions were treated as single mutations. Haplotype relationships were assessed using the median-joining network method^54^ in network 4.1.0.8 (http://www.fluxus-engineering.com).

We used the program haplonst^55^ to calculate the total and within-population haplotype diversity (*h*_T_ and *h*_S_), as well as population differentiation based on ordered (*N*_ST_) and unordered (*G*_ST_) haplotypes. The values of *N*_ST_ and *G*_ST_ were then compared using *U*-statistics to test for the presence of phylogeographic structure. To test the EI model for each species, we calculated the correlation of genetic variation with latitude by performing a multiple regression analysis in spss version 13.0 (SPSS Inc, Chicago, IL). Spatial genetic structure of each species was analyzed by the program samova (Spatial Analysis of Molecular Variation)^56^, which seeks for the best grouping (*K*) of populations that are geographically homogeneous and maximally differentiated from each other.

Tajima’s *D*^57^ and Fu’s *F*_S_^58^ were tested for each species to infer potential population growth and expansion. Mismatch distribution analyses^59^ were implemented in arlequin (ver. 3.1)^60^ under the model of demographic expansion. The goodness-of-fit was tested with the sum of squared deviations (*SDD*) between observed and expected mismatch distributions, and raggedness index (*H*_Rag_), using 1000 parametric bootstrap replicates. The expansion time (in generations) for expanding species or groups was calculated using the formula *T = τ/*2 *μkg*^59^, where *μ* is the substitution rate in substitution/site/year, *k* is the average sequence length used for analysis (*C. tibetana*: 1826 bp; *M. thunbergii*: 1450 bp; *S*. *superba*: 1399 bp), and *g* is the generation time in years. The *μ* of *C. tibetana* was set as 0.71 × 10^−9^ substitutions per site year^−1^ (s/s/y), which is the mean substitution rate of two chloroplast intergenic spacers (*ndh*J-*trn*F and *atp*I-*atp*H) in a closely related genus *Fagus* we previously studied^61^. This rate is similar to *μ* of three chloroplast intergenic spacers (*trn*H-*psb*A, *trn*T-*trn*L, and *atp*I-*atp*H) in another Fagaceae member, *Quercus glauca* (0.96 × 10^−9^ or 0.69 × 10^−9^ s/s/y)^15^. To approximate the substitution rate of chloroplast genomic non-coding regions in *M. thunbergii*,a phylogenetic tree of Lauraceae was reconstructed based on the chloroplast sequences (*rpl*16, *trn*L-F, and *psb*A-*trn*H) of species in Fig. 3 of Nie *et al*.^62^, and the substitution rates across the trees were computed by beast v.1.7.1 using the same settings as that study used. We chose the local substitution rate (4 × 10^−9^ s/s/y) of clade D of Fig. 3 in Nie *et al*.^62^ to represent the substitution rate of *psb*A*–trn*H and *rpl*32*–trn*L^(UAG)^ in *M. thunbergii*, because substitution rates varied among clades and clade D is a minimally monophyletic group that includes *M. thunbergii* when only chloroplast sequences were used for phylogenetic reconstruction. Similarly, we reconstructed the phylogenetic tree of Theaceae in Fig. 2 of Li *et al*.^63^ using beast v.1.7.1 to approximate the substitution rate of chloroplast genome in *S*. *superba.* The local substitution rate (0.6 × 10^−9^ s/s/y) of the clade C1 [see Fig. 2 in Li *et al*.^63^] in which the genus *Schima* was embedded, was adopted in this study. There is no accurate record for the first reproduction age of the three species. For *C. tibetana*, 25-year was used based on the generation time reported in another *Castanopsis* species^64^. In Lauraceae, *Ocotea tenera* takes at least 5 years to reach its reproductive maturity^65^, but the age at maturity of *Sassafras* was reported as 10 years^66^. Natural populations are likely to take a longer time to reach reproductive maturity. Therefore, we used 10 years as the generation time for *M. thunbergii*. We used 8 years to approximate the generation time of *S*. *superba* based on observations on age of first reproduction of cultivated trees at the arboretum of Jiangxi Agricultural University (Z.Y. Zhang, personal observation).

We used the Bayesian skyline plots (BSPs) method^67^ as implemented in beast v. 1.7.1 for estimating fluctuations in the effective population size of each species using the above substitution rates accordingly. MCMC chains were run for 20,000,000 generations for *C. tibetana*, *M. thunbergii* and *S*. *superba* under the GTR, GTR + G and HKY model chosen by jModelTest 2.1.5 respectively^68^.

### Species distribution modeling

We employed the maximum entropy approach (maxent)^69^ to predict the distribution of the three species at the present time and at the time of the LGM (0.021–0.018 Ma). Nineteen environmental variables for present and the time of the LGM (MIROC 3.2 scenario) were compiled from the WORLDCLIM database with a resolution of 2.5 arc-min (http://www.worldclim.org)^70^ for each environmental layer. To avoid over-fitting of niche models, seven variables with pairwise Pearson correlation coefficients *r* ≤ 0.70 (annual mean temperature, BIO_1_, mean diurnal range, BIO_2_, isothermality, BIO_3_, temperature seasonality, BIO_4_, annual precipitation, BIO_12_, precipitation seasonality, BIO_15_ and precipitation of coldest quarter, BIO_19_) were retained for subsequent analyses.

SDMs were constructed using 205/127/169 presence records of *Castanopsis tibetana*, *Machilus thunbergii*, and *Schima superba*, respectively, including all sites recorded in the field work of this study and for which specimen records with GPS (Chinese Virtual Herbarium) data are available. Each model was run 10 times using the default parameters (convergence threshold of 10^−5^, maximum iterations of 500 and regularization multiplier of 1) and the following user-selected features: application of a random seed, duplicate presence records removal and logistic probabilities used for the output. 80% of species records were used to train the model and 20% to test the model. Model accuracy was measured by the area under the ‘Receiver Operating Characteristic (ROC) Curve’ (AUC)^71^,^72^. A score between 0.7 and 1.0 indicates that the model performs better than random and was considered acceptable discrimination^71^.

We then measured niche differences between each species’ ENM following the suggestions of Warren *et al*.^73^. enmtools, version 1.3, was used to calculate Schoener’s D^74^ and a standardized version of Hellinger distance (calculated as I)^73^. Both D and I ranged from 0 (no niche overlap) to 1 (identical niches). Next, we conducted an identity test to build niche models based on a set of pseudoreplicates generated from a random sampling of data points pooled for each pair of species. A total of 100 replicates were used to generate the pseudoreplicated data sets. The observed measures of niche similarity between species were compared with the null distribution.

## Additional Information

**How to cite this article**: Fan, D. *et al*. Idiosyncratic responses of evergreen broad-leaved forest constituents in China to the late Quaternary climate changes. *Sci. Rep.* **6**, 31044; doi: 10.1038/srep31044 (2016).

## Supplementary Material

Supplementary Information

## Figures and Tables

**Table 1:** Mismatch distribution analysis (MDA) of cpDNA sequence data for three broadleaved evergreen tree species.

Regions	*τ*	*t* (y)	Observed modality of mismatch distribution	*SSD*	*P*	*H*_Rag_	*P*
*C. tibetana*	0.625 (0–3.438)	9,600 (0–53,000)	unimodal	0.023	0.251	0.040	0.640
*M. thunbergii*	0.438 (0–3.828)	3,700 (0–33,000)	unimodal	0.009	0.544	0.042	0.822
*S. superba*	0.082 (0–1.458)	NC	bimodal	0.188	0.040	0.491	0.020
*west*	0.082 (0–1.547)	6,100 (0–115,100)	unimodal	0.051	0.231	0.178	0.517
*east*	0.844 (0.717–1.045)	NC	bimodal	0.017	0.003	0.146	0

Goodness-of-fit of observed to theoretical mismatch distributions under a demographic expansion model^60^ are tested with the sum of squared deviations (*SSD*) and raggedness index (*H*_Rag_). Upper and lower 95% confidence limits around estimates of *τ*, and associated ranges of *t* (in years bp) are in parentheses.

NC, not calculated.

## References

[b1] Song Y. C. The essential characteristics and main types of the broad-leaved evergreen forest in China. Phytocoenologia 16, 105–123 (1988).

[b2] Wang X. H., Kent M. & Fang X. F. Evergreen broad-leaved forest in Eastern China: its ecology and conservation and the importance of resprouting in forest restoration. Forest. Ecol. Manag. 245, 76–87 (2007).

[b3] Zhao D., Wu S. & Yin Y. Responses of terrestrial ecosystems’ net primary productivity to future regional climate change in China. PloS One 8, e60849 (2013). 23593325 10.1371/journal.pone.0060849 PMC3623914

[b4] Pearman P. B. . Prediction of plant species distributions across six millennia. Ecol. Lett. 11, 357–369 (2008). 18279357 10.1111/j.1461-0248.2007.01150.x

[b5] Gavin D. G. . Climate refugia: joint inference from fossil records, species distribution models and phylogeography. New Phylot. 204, 37–54 (2014). 10.1111/nph.12929 25039238

[b6] Riddle B. R. The molecular phylogeographic bridge between deep and shallow history in continental biotas. Trends Ecol. Evol. 11, 207–211 (1996). 21237810 10.1016/0169-5347(96)10032-x

[b7] Hewitt G. M. The genetic legacy of the Quaternary ice ages. Nature 405, 907–913 (2000). 10879524 10.1038/35016000

[b8] Zheng Z., Yuan B. Y. & Petit-Maire N. Palaeoenvironments in China during the Last Glacial Maximum and the Holocene Optimum. Episodes 21, 152–158 (1998).

[b9] Yu G. . Palaeovegetation of China: a pollen data-based synthesis for the mid-Holocene and last glacial maximum. J. Biogeogr. 27, 635–666 (2000).

[b10] Harrison S. P., Yu G., Takahara H. & Prentice I. C. Palaeovegetation diversity of temperate plants in East Asia. Nature 413, 129–130 (2001). 11557970 10.1038/35093166

[b11] Ni J., Cao X., Jeltsch F. & Herzschuh U. Biome distribution over the last 22,000 yr in China. Palaeogeogr. Palaeoclim. Palaeoecol. 409, 33–47 (2014).

[b12] Qian H. & Ricklefs R. E. Palaeovegetation: diversity of temperate plants in East Asia–reply. Nature 413, 130 (2001). 10.1038/35093166 11557970

[b13] Shi M. M., Michalski S. G., Welk E., Chen X. Y. & Durka W. Phylogeography of a widespread Asian subtropical tree: genetic east-west differentiation and climate envelope modelling suggest multiple glacial refugia. J. Biogeogr. 41, 1710–1720 (2014).

[b14] Sun Y., Hu H. Q., Huang H. W. & Vargas-Mendoza C. F. Chloroplast diversity and population differentiation of *Castanopsis fargesii* (Fagaceae): a dominant tree species in evergreen broad-leaved forest of subtropical China. Tree Genet. Genomes 10, 1531–1539 (2014).

[b15] Xu J. . Phylogeography of *Quercus glauca* (Fagaceae), a dominant tree of East Asian subtropical evergreen forests, based on three chloroplast DNA interspace sequences. Tree Genet. Genomes 11, 805 (2015).

[b16] Webb C. O., Ackerly D. D., McPeek M. A. & Donoghue M. J. Phylogenies and community ecology. Annu. Rev. Ecol. Syst. 33, 475–505 (2002).

[b17] Wiens J. J. & Graham C. H. Niche conservatism: integrating evolution, ecology, and conservation biology. Annu. Rev. Ecol. Evol. Syst. 36, 519–539 (2005).

[b18] Wang J., Gao P. X., Kang M., Lowe A. J. & Huang H. W. Refugia within refugia: the case study of a canopy tree *Eurycorymbus cavaleriei* in subtropical China. J. Biogeogr. 36, 2156–2164 (2009).

[b19] Li Y., Yan H. F. & Ge X. J. Phylogeographic analysis and environmental niche modelling of widespread shrub *Rhododendron simsii* in China reveals multiple glacial refugia during the last glacial maximum. J. Syst. Evol. 50, 362–373 (2012).

[b20] Wang Y. H. . Molecular phylogeography and ecological niche modelling of a widespread herbaceous climber, *Tetrastigma hemsleyanum* (Vitaceae): insights into Plio-Pleistocene range dynamics of evergreen forest in subtropical China. New Phytol. 206, 852–867 (2015). 25639152 10.1111/nph.13261

[b21] López-Pujol J., Zhang F. M., Sun H. Q., Ying T. S. & Ge S. Centres of plant endemism in China: places for survival or for speciation? J. Biogeogr. 38, 1267–1280 (2011).

[b22] Arbogast B. S. & Kenagy G. J. Comparative phylogeography as an integrative approach to historical biogeography. J. Biogeogr. 28, 819–825 (2001).

[b23] Avise J. C. Phylogeography: retrospect and prospect. J. Biogeogr. 36, 3–15 (2009).

[b24] Hewitt G. M. Postglacial recolonization of European biota. Biol. J. Linn. Soc. 68, 87–112 (1999).

[b25] Lapointe F. J. & Rissler L. J. Congruence, consensus, and the comparative phylogeography of codistributed species in California. Amer. Natur. 166, 290–299 (2005). 16032580 10.1086/431283

[b26] Soltis D. E., Morris A. B., Mclachlan J. S., Manos P. S. & Soltis P. S. Comparative phylogeography of unglaciated eastern North America. Mol. Ecol. 15, 4261–4293 (2006). 17107465 10.1111/j.1365-294X.2006.03061.x

[b27] Carstens B., Brunsfeld S. J., Demboski J. R., Good J. & Sullivan J. Investigating the evolutionary history of the Pacific Northwest mesic forest ecosystem: hypothesis testing within a comparative phylogeographic framework. Evolution 59, 1639–1652 (2005). 16331838

[b28] Sunnucks P. . A tale of two flatties: different responses of two terrestrial flatworms to past environmental climatic fluctuations at Tallaganda in montane southeastern Australia. Mol. Ecol. 15, 4513–4531 (2006). 17107480 10.1111/j.1365-294X.2006.03107.x

[b29] Moussalli A., Moritz C., Williams S. E. & Carnaval A. C. Variable responses of skinks to a common history of rainforest fluctuation: concordance between phylogeography and palaeo-distribution models. Mol. Ecol. 18, 483–499 (2009). 19161469 10.1111/j.1365-294X.2008.04035.x

[b30] Svenning J. C., Flójgaard C., Marske K. A. & Nógues-Bravo D. Applications of species distribution modelling to palaeobiology. Quat. Sci. Rev. 30, 2930–2947 (2011).

[b31] Wu C. Y. Vegetation of China. (Science Press, Beijing, 1980).

[b32] Crisp M. D. . Phylogenetic biome conservatism on a global scale. Nature 458, 754–756 (2009). 19219025 10.1038/nature07764

[b33] Hewitt G. M. Some genetic consequences of ice ages, and their role in divergence and speciation. Biol. J. Linn. Soc. 58, 247–276 (1996).

[b34] Wu S. H. . Contrasting phylogeographical patterns of two closely related species, *Machilus thunbergii* and *Machilus kusanoi* (Lauraceae), in Taiwan. J. Biogeogr. 33, 936–947 (2006).

[b35] Liao P. C. . Historical spatial range expansion and a very recent bottleneck of *Cinnamomum kanehirae* Hay. (Lauraceae) in Taiwan inferred from nuclear genes. BMC Evol. Biol. 10, 124 (2010). 20433752 10.1186/1471-2148-10-124 PMC2880300

[b36] Liu J. Q., Sun Y. S., Ge X. J., Gao L. M. & Qiu Y. X. Phylogeographic studies of plants in China: Advances in the past and directions in the future. J. Syst. Evol. 50, 267–275 (2012).

[b37] Li X. H., Shao J. W., Lu C., Zhang X. P. & Qiu Y. X. Chloroplast phylogeography of a temperate tree *Pteroceltis tatarinowii* (Ulmaceae) in China. J. Syst. Evol. 50, 325–333 (2012).

[b38] Tian S. . Repeated range expansions and inter-/postglacial recolonization routes of *Sargentodoxa cuneata* (Oliv.) Rehd. et Wils. (Lardizabalaceae) in subtropical China revealed by chloroplast phylogeography. Mol. Phylogenet. Evol. 85, 238–246 (2015). 25732070 10.1016/j.ympev.2015.02.016

[b39] Cun Y. Z. & Wang X. Q. Phylogeography and evolution of three closely related species of *Tsuga* (hemlock) from subtropical eastern Asia: further insights into speciation of conifers. J. Biogeogr. 42, 315–327 (2015).

[b40] Gutiérrez-García T. A. & Vázquez-Domínguez E. Comparative phylogeography: designing studies while surviving the process. Bioscience 61, 857–868 (2011).

[b41] Williams J. W. & Shuman B. N., Webb lll, T., Bartlein, P.J. & Leduc, P.L. Late-Quaternary vegetation dynamics in north America: scaling from taxa to biomes. Ecol. Monogr. 74, 309–334 (2004).

[b42] Morris A. B., Graham C. H., Soltis D. E. & Soltis P. S. Reassessment of phylogeographical structure in an eastern North American tree using Monmonier’s algorithm and ecological nich modelling. J. Biogeogr. 37, 1657–1667 (2010).

[b43] Rodríguez-Sánchez F., Hampe A., Jordano P. & Arroyo J. Past tree range dynamics in the Iberian Peninsula inferred through phylogeography and palaeodistribution modelling: a review. Rev. Palaeobot. Palyno. 162, 507–521 (2010).

[b44] Prentice I. C., Guiot J., Huntley B., Jolly D. & Cheddadi R. Reconstructing biomes from palaeoecological data: a general method and its application to European pollen data at 0 and 6 ka. Clim. Dynam. 12, 185–194 (1996).

[b45] Wiens J. A., Stralberg D., Jongsomjit D., Howell C. A. & Snyder M. A. Niches, models, and climate change: assessing the assumptions and uncertainties. Proc. Nat. Acad. Sci. USA 106, 19729–19736 (2009). 19822750 10.1073/pnas.0901639106 PMC2780938

[b46] Stewart J. R., Lister A. M., Barnes I. & Dalén L. Refugia revisited: individualistic responses of species in space and time. Proc. Roy. Soc. London. Ser. B, Biol. Sci. 277, 6615–6671 (2010). 10.1098/rspb.2009.1272 PMC2842738 19864280

[b47] Hampe A. & Jump A. S. Climate relicts: past, present, future. Annu. Rev. Ecol. Evol. Syst. 42, 313–333 (2011).

[b48] Castro-Díez P., Villar-Salvador P., Pérez-Rontomé C., Maestro-Martínez M. & Montserrat-Martí G. Leaf morphology and leaf chemical composition in three *Querus* (Fagaceae) species along a rainfall gradient in NE Spain. Trees 11, 127–134 (1997).

[b49] Zhang G. S. Study on the intraspecific and interspecific competition in *Machilus thunbergii* community. J. Fujian Coll. For. 30, 179–182 (2010).

[b50] Dullinger S. . Extinction debt of high-mountain plants under twenty-first-century climate change. Nat. Clim. Change 2, 619–622 (2012).

[b51] Saeki I., Dick C. W., Barnes B. V. & Murakami N. Comparative phylogeography of red maple (*Acer rubrum* L.) and silver maple (*Acer saccharinum* L.): impacts of habitat specialization, hybridization and glacial history. J. Biogeogr. 38, 992–1005 (2011).

[b52] Doyle J. J. & Doyle J. L. A rapid DNA isolation procedure for small quantities of fresh leaf tissue. Phytochemistry 19, 11–15 (1987).

[b53] Thompson J. D., Gibson T. J., Plewniak F., Jeanmougin F. & Higgins D. G. The CLUSTAL_X windows interface: flexible strategies for multiple sequence alignment aided by quality analysis tools. Nucl. Acids Res. 25, 4876–4882 (1997). 9396791 10.1093/nar/25.24.4876 PMC147148

[b54] Bandelt H. J., Forster P. & Rohl A. Median-joining networks for inferring intraspecific phylogenies. Mol. Biol. Evol. 16, 37–48 (1999). 10331250 10.1093/oxfordjournals.molbev.a026036

[b55] Pons O. & Petit R. J. Measuring and testing genetic differentiation with ordered versus unordered alleles. Genetics 144, 1237–1245 (1996). 8913764 10.1093/genetics/144.3.1237 PMC1207615

[b56] Dupanloup I., Schneider S. & Excoffier L. A simulated annealing approach to define the genetic structure of populations. Mol. Ecol. 11, 2571–2581 (2002). 12453240 10.1046/j.1365-294x.2002.01650.x

[b57] Tajima F. Statistical method for testing the neutral mutation hypothesis by DNA polymorphism. Genetics 123, 585–595 (1989). 2513255 10.1093/genetics/123.3.585 PMC1203831

[b58] Fu Y. X. & Li W. H. Statistical tests of neutrality of mutations. Genetics 133, 693–709 (1993). 8454210 10.1093/genetics/133.3.693 PMC1205353

[b59] Rogers A. R. & Harpending H. C. Population growth makes waves in the distribution of pairwise genetic differences. Mol. Biol. Evol. 9, 552–569 (1992). 1316531 10.1093/oxfordjournals.molbev.a040727

[b60] Excoffier L., Laval G. & Schneider S. Arlequin ver. 3.1: an integrated software package for population genetics data analysis. Evol. Bioinformatics Online 1, 47–50 (2005). PMC2658868 19325852

[b61] Zhang Z. Y. . Comparative phylogeography of two sympatric beeches in subtropical China: Species-specific geographic mosaic of lineages. Ecol. Evol. 3, 4461–4472 (2012). 24340187 10.1002/ece3.829 PMC3856746

[b62] Nie N. L., Wen J. & Sun H. Phylogeny and biogeography of *Sassafras* (Lauraceae) disjunct between eastern Asia and eastern North America. Plant Syst. Evol. 267, 191–203 (2007).

[b63] Li M. M., Li J. H., Del Tredici P., Corajod J. & Fu C. X. Phylogenetics and biogeography of Theaceae based on sequences of plastid genes. J. Syst. Evol. 51, 396–404 (2013).

[b64] Liu R. L., Wang L. & Du T. Z. Features of population ecological quantity field of *Castanopsis fabri* community in Jinggang Mountain. Sci. Silv. Sin. 44, 1–7 (2008).

[b65] Wheelwright N., Sinclair J. P. & Hochwender C. Leaf size in three generations of a dioecious tropical tree, *Ocotea tenera* (Lauraceae): sexual dimorphism and changes with age. Am. J. Bot. 99, 1350–1355 (2012). 22847542 10.3732/ajb.1200182

[b66] Verdú M. Age at maturity and diversification in woody angiosperms. Evolution 56, 1352–1361 (2002). 12206237 10.1111/j.0014-3820.2002.tb01449.x

[b67] Drummond A. J., Rambaut A., Shapiro B. & Pybus O. G. Bayesian coalescent inference of past population dynamics from molecular sequences. Mol. Biol. Evol. 22, 1185–1192 (2005). 15703244 10.1093/molbev/msi103

[b68] Darriba D., Taboada G. L., Doallo R. & Posada D. jModelTest 2: more models, new heuristics and parallel computing. Nat. Meth. 9, 772 (2012). 10.1038/nmeth.2109 PMC4594756 22847109

[b69] Phillips S. J., Anderson R. P. & Schapire R. E. Maximum entropy modelling of species geographic distributions. Ecol. Model. 190, 231–259 (2006).

[b70] Hijmans R. J., Cameron S. E., Parra J. L., Jones P. G. & Jarvis A. Very high resolution interpolated climate surfaces for global land areas. Int. J. Climatol. 25, 1965–1978 (2005).

[b71] Fielding A. H. & Bell J. F. A review of methods for the assessment of prediction errors in conservation presence/absence models. Environ. Conserv. 24, 38–49 (1997).

[b72] Elith J. . Novel methods improve prediction of species’ distributions from occurrence data. Ecography 29, 129–151 (2006).

[b73] Warren D. L., Glor R. E. & Turelli M. Environmental niche equivalency versus conservatism: quantitative approaches to niche evolution. Evolution 62, 2868–2883 (2008). 18752605 10.1111/j.1558-5646.2008.00482.x

[b74] Schoener T. W. The Anolis lizards of Bimini: resource partitioning in a complex fauna. Ecology 49, 704–726 (1968).

