# Supplementary material for: Idiosyncratic responses of evergreen broad-leaved forest constituents in China to the late Quaternary climate changes
Source: Sci Rep. 2016 Aug 18;6:31044. doi: 10.1038/srep31044 (PMC4989166; doi:10.1038/srep31044)
Supplement: Supplementary Information [file srep31044-s1.doc]

**Supplementary Information**

**Idiosyncratic responses of evergreen broad-leaved forest constituents in China to the late Quaternary climate changes**

Dengmei fan^1¶^, Wan Hu^1¶^, Bo Li^1^, Ashley B. Morris^2^, Min Zheng^1^, Douglas E. Soltis^3,4^, Pamela S. Soltis^3^ and Zhiyong zhang^1^*

^1^ Laboratory of Subtropical Biodiversity, Jiangxi Agricultural University, Nanchang, Jiangxi, 330045, China. ^2^Department of Biology, Middle Tennessee State University, Murfreesboro, TN 37132, Tennessee, USA. ^3^Department of Biology, University of Florida, Gainesville, FL 17 32611, USA. ^4^Florida Museum of Natural History, University of Florida, Gainesville, FL 17 32611, USA.

*correspondence: Zhi-Yong Zhang, Email: [pinus-rubus@163.com;](mailto:pinus-rubus@163.com;)

**^¶^These authors contributed equally to this work.**

Table S1. Previous phylogeograhic and species distribution modelling (SDM) studies on EBLF plant species.

| Life form/Taxon | phylogeographic result | SDM result |
| --- | --- | --- |
| **evergreen broadleaved tree** |  |  |
| *Castanopsis eyrei*^1^ | ISS model | ISS model |
| *Castanopsis fargesii*^2^ | ISS model | — |
| *Castanopsis hystrix*^3^ | ISS model | — |
| *Loropetalum chinense*^4^ | ISS model | ISS model |
| *Quercus glauca*^5^ | ISS model | ISS model |
| **deciduous broadleaved tree** |  |  |
| *Cercidiphyllum japonicum*^6^ | ISS model | ISS model |
| *Cyclocarya paliurus*^7^ | ISS model | — |
| *Emmenopterys henryi*^8^ | ISS model | ISS model |
| *Euptelea*^9^ | ISS model | ISS model |
| *Eurycorymbus cavaleriei*^10^ | ISS model | — |
| *Fagus longipetiolata*^11^ | ISS model | — |
| *Pteroceltis tatarinowii*^12^ | EI model | — |
| *Tetracentron sinense*^13^ | ISS model | — |
| *Quercus variabilis*^14^ | ISS model | — |
| **semi-evergreen shrub** |  |  |
| *Rhododendron simsii*^15^ | ISS model | — |
| **conifer** |  |  |
| *Cathaya argyrophylla*^16^ | ISS model | — |
| *Pinus kwangtungensis*^17^ | ISS model | — |
| *Taxus wallichiana*^18^ | ISS model | — |
| **perennial deciduous climber** |  |  |
| *Sargentodoxa cuneata*^19^ | EI model | — |
| *Tetrastigma hemsleyanum*^20^ | ISS model | ISS model |

Table S2. Details of sampled populations of *Castanopsis tibetana*, *Machilus thunbergii* and *Schima superba* from subtropical China. Each species’ column shows number of each haplotype, haplotype diversity (*h*) and nucleotide diversity (π) per population.

| Provinces/ Population ID | sampling sites | Latitude  (°N) | Longitude  (°E) | *Castanopsis tibetana* | |  |  | *Machilus thunbergii* | |  | | *Schima superba* | |  |
| --- | --- | --- | --- | --- | --- | --- | --- | --- | --- | --- | --- | --- | --- | --- |
|  |  |  |  | Haplotype | *h* | π |  | Haplotype | *h* | π |  | Haplotype | *h* | π |
| Hunan (HN) |  |  |  |  |  |  |  |  |  |  |  |  |  |  |
| 1 | Huitong | 26.87 | 109.72 | CH1(8) | 0 | 0 |  | MH5(10) | 0 | 0 |  | SH8(4), SH6(6) | 0.533 | 0.00038 |
| 2 | Suining | 26.43 | 110.16 | CH1(3),CH16(3) | 0.600 | 0.00173 |  | MH12(4), MH13(5) | 0.556 | 0.00039 |  | SH8(5) | 0 | 0 |
| 3 | Shuangpai | 26.09 | 111.93 | — | — | — |  | MH2(7) | 0 | 0 |  | — | — | — |
| 4 | Yizhang | 24.97 | 112.95 | CH1(1), CH6(7) | 0.250 | 0.00014 |  | MH4(3), MH5(7) | 0.467 | 0.00033 |  | SH8(11) | 0 | 0 |
| 5 | Hengshan | 27.24 | 112.86 | CH6(8) | 0 | 0 |  | MH4(6) | 0 | 0 |  | SH8(2), SH9(5) | 0.476 | 0.00034 |
| 6 | Dong'an | 26.41 | 111.31 | CH6(8) | 0 | 0 |  | — | — | — |  | SH8(1) | 0 | 0 |
| 7 | Hupingshan | 30.01 | 110.62 | CH7(5), CH1(1) | 0.333 | 0.00019 |  | — | — | — |  | — | — | — |
| 8 | Sangzhi | 29.75 | 110.05 | CH7(8) | 0 | 0 |  | — | — | — |  | — | — | — |
| 9 | Guzhang | 28.66 | 110.08 | CH7(7) | 0 | 0 |  | — | — | — |  | — | — | — |
| 10 | Liuyang | 28.43 | 114.07 | CH1(5) | 0 | 0 |  | — | — | — |  | SH8(7), SH9(2) | 0.389 | 0.00028 |
| Jiangxi (JX) |  |  |  |  |  |  |  |  |  |  |  |  |  |  |
| 11 | Fenyi | 27.66 | 114.60 | CH1(4) | 0 | 0 |  | MH10(8) | 0 | 0 |  | SH8(2), SH9(8) | 0.356 | 0.00025 |
| 12 | Ningdu | 26.92 | 115.91 | CH2(9) | 0 | 0 |  | — | — | — |  | SH8(9) | 0 | 0 |
| 13 | Guanshan | 28.53 | 114.73 | CH1(9) | 0 | 0 |  | — | — | — |  | — | — | — |
| 14 | Yongxiu | 29.08 | 115.61 | — | — | — |  | MH4(9) | 0 | 0 |  | — | — | — |
| 15 | Lichuan | 27.12 | 116.88 | CH1(9),CH11(1) | 0.200 | 0.00011 |  | MH4(13) | 0 | 0 |  | SH5(9) | 0 | 0 |
| 16 | Yanshan | 28.11 | 117.70 | — | — | — |  | MH4(9) | 0 | 0 |  | SH5(9), SH8(1) | 0.200 | 0.00072 |
| 17 | Xiushui | 28.84 | 114.72 | CH1(7) | 0 | 0 |  | MH4(10) | 0 | 0 |  | SH8(1) | 0 | 0 |
| 18 | Sanqingshan | 28.91 | 118.06 | CH6(9) | 0 | 0 |  | MH4(10) | 0 | 0 |  | SH5(11) | 0 | 0 |
| 19 | Zixi | 27.76 | 117.18 | CH6(1),CH11(9) | 0.200 | 0.00023 |  | MH8(10) | 0 | 0 |  | SH5(10) | 0 | 0 |
| 20 | Sanbaishan | 25.15 | 115.42 | — | — | — |  | — | — | — |  | SH2(11) | 0 | 0 |
| 21 | Qilushan | 25.69 | 115.30 | — | — | — |  | — | — | — |  | SH2(11), SH9(1) | 0.167 | 0.00084 |
| 22 | Wanzai | 28.35 | 114.51 | — | — | — |  | — | — | — |  | SH8(12) | 0 | 0 |
| 23 | Fuliang | 29.55 | 117.66 | — | — | — |  | MH4(5) | 0 | 0 |  | SH5(8) | 0 | 0 |
| 24 | Dazhangshan | 29.18 | 117.74 | CH18(6) | 0 | 0 |  | — | — | — |  | — | — | — |
| 25 | Chongyi | 25.62 | 114.32 | — | — | — |  | MH4(7), MH6(3) | 0.467 | 0.00065 |  | SH8(6), SH9(4) | 0.533 | 0.00038 |
| 26 | Ruijin | 25.92 | 115.97 | CH14(5), CH13(2) | 0.476 | 0 |  | — | — | — |  | SH2(11) | 0 | 0 |
| 27 | Dingnan | 24.90 | 115.16 | CH1(5) | 0 | 0 |  | MH4(10) | 0 | 0 |  | SH2(10) | 0 | 0 |
| 28 | Quannan | 24.93 | 114.58 | CH6(4), CH5(2) | 0 | 0 |  | — | — | — |  | SH2(7), SH8(1), SH9(1) | 0.417 | 0.00184 |
| 29 | Le'an | 27.26 | 116.13 | CH8(5) | 0 | 0 |  | MH4(10) | 0 | 0 |  | SH9(10) | 0 | 0 |
| 30 | Shicheng | 26.02 | 116.34 | — | — | — |  | MH4(4), MH5(2) | 0.533 | 0.00037 |  | — | — | — |
| 31 | Fengxin | 28.62 | 114.94 | CH1(6) | 0 | 0 |  | — | — | — |  | — | — | — |
| 32 | Jinggangshan | 26.53 | 114.14 | CH1(2), CH15(6) | 0.429 | 0.00074 |  | MH5(9) | 0 | 0 |  | SH8(6), SH9(4) | 0.533 | 0.00038 |
| 33 | Taihe | 26.53 | 115.06 | CH6(2) | 0 | 0 |  | MH4(13) | 0 | 0 |  | SH2(1), SH8(7), SH9(2) | 0.511 | 0.00112 |
| 34 | Ganxian | 25.74 | 115.18 | CH1(3), CH4(3), CH3(1) | 0.286 | 0.00033 |  | MH7(10) | 0 | 0 |  | — | — | — |
| 35 | Tonggu | 28.66 | 114.30 | CH6(7) | 0 | 0 |  | MH4(10) | 0 | 0 |  | — | — | — |
| 36 | Lushan | 29.55 | 116.01 | CH6(3) | 0 | 0 |  | MH4(9) | 0 | 0 |  | — | — | — |
| Zhejiang (ZJ) | |  |  |  |  |  |  |  |  |  |  |  |  |  |
| 37 | Qingliangfeng | 30.17 | 119.20 | — | — | — |  | MH4(7) | 0 | 0 |  | SH4(10) | 0 | 0 |
| 38 | Jiulongshan | 28.47 | 118.88 | CH1(5), CH12(3) | 0.536 | 0.00061 |  | MH4(9) | 0 | 0 |  | SH5(9), SH8(1) | 0.200 | 0.00072 |
| 39 | Tiantai | 29.26 | 121.10 | — | — | — |  | MH4(11) | 0 | 0 |  | SH5(10) | 0 | 0 |
| 40 | TianMushan | 30.37 | 119.47 | — | — | — |  | MH4(10) | 0 | 0 |  | — | — | — |
| 41 | Qingyuan | 27.75 | 119.21 | — | — | — |  | MH4(3) | 0 | 0 |  | SH5(10) | 0 | 0 |
| 42 | Jinzifeng | 27.68 | 119.01 | CH6(8) | 0 | 0 |  | MH4(10) | 0 | 0 |  | — | — | — |
| 43 | Pan'an | 28.98 | 120.52 | — | — | — |  | MH4(7) | 0 | 0 |  | SH5(10) | 0 | 0 |
| 44 | Yuyao | 29.74 | 121.11 | — | — | — |  | — | — | — |  | SH5(10) | 0 | 0 |
| Fujian (FJ) |  |  |  |  |  |  |  |  |  |  |  |  |  |  |
| 45 | Jiangle | 26.52 | 117.30 | CH1(2), CH12(8) | 0.356 | 0.00041 |  | MH4(7) | 0 | 0 |  | SH5(10) | 0 | 0 |
| 46 | Yongchun | 25.32 | 118.29 | — | — | — |  | MH11(2), MH4(4), MH6(2) | 0.714 | 0.0013 |  | SH2(10) | 0 | 0 |
| 47 | Wuyishan | 27.75 | 117.68 | CH11(10) | 0 | 0 |  | MH4(3) | 0 | 0 |  | — | — | — |
| 48 | Shaxian | 26.52 | 117.93 | — | — | — |  | — | — | — |  | SH5(7), SH3(3) | 0.467 | 0.00067 |
| 49 | Wuping | 25.16 | 116.15 | CH10(10) | 0 | 0 |  | MH3(6), MH4(1), MH6(3) | 0.600 | 0.00102 |  | SH2(5), SH5(5) | 0.556 | 0.0004 |
| 50 | Sanming | 26.21 | 117.56° | — | — | — |  | — | — | — |  | SH2(10), SH1(1) | 0.182 | 0.00013 |
| 51 | Yong'an | 25.87 | 117.43 | CH12(6), CH11(2) | 0.429 | 0.00024 |  | MH4(1), MH6(7) | 0.250 | 0.00035 |  | SH2(11) | 0 | 0 |
| 52 | Nanjing | 24.52 | 117.26 | — | — | — |  | MH4(4), MH6(2),MH1(1)  1HHHMH1(1) | 0.667 | 0.00087 |  | SH2(9), SH5(1) | 0.2 | 0.00014 |
| 53 | Shangkang | 25.34 | 116.74 | — | — | — |  | MH4 (1), MH6 (10) | 0.182 | 0.00025 |  | SH2(10) | 0 | 0 |
| 54 | Nanping | 26.63 | 118.26 | CH12(1) | 0 | 0 |  | MH4(8) | 0 | 0 |  | SH2(4), SH3(6) | 0.533 | 0.00038 |
| 55 | Dehua | 25.68 | 118.19 | CH1(3), CH12(3) | 0.600 | 0.00069 |  | MH4(2), MH6(1), MH5(1) | 0.667 | 0.00047 |  | SH2(10) | 0 | 0 |
| Guangdong (GD) | |  |  |  |  |  |  |  |  |  |  |  |  |  |
| 56 | Boluo | 23.27 | 114.06 | — | — | — |  | MH11(1), MH9(7), MH3(2) | 0.511 | 0.0011 |  | SH2(10) | 0 | 0 |
| 57 | Xinyi | 22.29 | 111.26 | — | — | — |  | MH5(12) | 0 | 0 |  | SH2(6), SH8(4) | 0.533 | 0.0023 |
| 58 | Zhaoqing | 23.17 | 112.52 | — | — | — |  | — | — | — |  | SH2(10) | 0 | 0 |
| 59 | Fengshun | 23.85 | 116.32 | — | — | — |  | MH6(10) | 0 | 0 |  | SH2(10) | 0 | 0 |
| 60 | Yingde | 24.19 | 113.41 | — | — | — |  | MH6(1), MH5(3) | 0.667 | 0.00047 |  | SH2(10) | 0 | 0 |
| 61 | Lianzhou | 24.87 | 112.64 | — | — | — |  | MH9(1), MH6(3), MH7(1) | 0.700 | 0.00071 |  | SH8(10) | 0 | 0 |
| Hubei (HB) |  |  |  |  |  |  |  |  |  |  |  |  |  |  |
| 62 | Xuan'en | 30.03 | 109.72 | CH17(4) | 0 | 0 |  | — | — | — |  | — | — | — |
| 63 | En'shi | 30.03 | 109.10 | CH17(10) | 0 | 0 |  | — | — | — |  | — | — | — |
| 64 | Lichuan | 30.24 | 108.82 | — | — | — |  | — | — | — |  | SH4(10) | 0 | 0 |
| Guangxi (GX) | |  |  |  |  |  |  |  |  |  |  |  |  |  |
| 65 | Lingui | 25.55 | 110.16 | CH1(9) | 0 | 0 |  | — | — | — |  | SH8(10) | 0 | 0 |
| 66 | Hezhou | 24.64 | 111.51 | — | — | — |  | — | — | — |  | SH6(10) | 0 | 0 |
| 67 | Dayaoshan | 23.97 | 110.13 | CH11(2), CH9(4) | 0.533 | 0.00061 |  | — | — | — |  | SH8(6), SH7(1) | 0.286 | 0.0002 |
| 68 | Mao'er'shan | 25.89 | 110.37 | CH6(10) | 0 | 0 |  | MH4(1), MH5(6), MH12(3) | 0.600 | 0.00047 |  | SH6(10) | 0 | 0 |
| 69 | Jinzhongshan | 24.37 | 104.57 | — | — | — |  | MH5(10) | 0 | 0 |  | — | — | — |
| Guizhou (GZ) | |  |  |  |  |  |  |  |  |  |  |  |  |  |
| 70 | Suiyang | 28.08 | 107.28 | CH1(10) | 0 | 0 |  | — | — | — |  | — | — | — |
| 71 | Leishan | 26.39 | 108.20 | CH1(10) | 0 | 0 |  | — | — | — |  | SH8(10) | 0 | 0 |
| 72 | Fanjingshan | 27.93 | 108.79 | CH1(3), CH19(7) | 0.467 | 0.0008 |  | — | — | — |  | — | — | — |
| 73 | Rongjiang | 25.77 | 108.33 | CH1(8) | 0 | 0 |  | — | — | — |  | SH8(11) | 0 | 0 |
| 74 | Liping | 26.24 | 109.31 | CH1(10) | 0 | 0 |  | MH12(5) | 0 | 0 |  | SH8(9) | 0 | 0 |
| Total |  |  |  |  | 0.797 | 0.00104 |  |  | 0.644 | 0.00079 |  |  | 0.765 | 0.00219 |

—, not sampled.

Table S3. Variable sites of the *rpl*32*–trn*L and *psb*A*–trn*H sequences for each of the haplotypes identified in *Castanopsis tibetana*, *Machilus thunbergii* and *Schima superba*, respectively.

| *Castanopsis tibetana* | | | | | | | | | | | | | | | | | | | | | | | | | | | | | | |
| --- | --- | --- | --- | --- | --- | --- | --- | --- | --- | --- | --- | --- | --- | --- | --- | --- | --- | --- | --- | --- | --- | --- | --- | --- | --- | --- | --- | --- | --- | --- |
|  | *rpl32–trnL* | | | | | | | | | | | | | | | | |  | *psbA–trnH* | | | | | | | | | | | |
|  | 7 | 7 | 2 |  | 2 | 5 |  | 7 | 7 |  |  |  |  | 1 |  |  | 1 |  | 1 | 1 | 1 |  | 1 | 1 | 1 | 1 | 1 |  | 1 | 1 |
| Haplotype | 2 | 3 | 2 | 235–255 | 6 | 0 | 677–699 | 2 | 7 | 925–929 | 1009–1020 | 1062–1076 | 1077–1081 | 0 | 1147–1151 | 1190–1197 | 2 |  | 3 | 3 | 4 | 1412–1418 | 4 | 4 | 4 | 5 | 5 | 1637–1645 | 6 | 6 |
|  |  |  | 9 |  | 7 | 1 |  | 9 | 4 |  |  |  |  | 9 |  |  | 1 |  | 2 | 9 | 0 |  | 4 | 9 | 9 | 0 | 2 |  | 5 | 8 |
|  |  |  |  |  |  |  |  |  |  |  |  |  |  | 7 |  |  | 1 |  | 9 | 2 | 5 |  | 0 | 5 | 6 | 7 | 5 |  | 4 | 3 |
| CH1 | T | T | T | – | G | A | – | T | G | 3^C^ | – | – | – | C | 7^C^ | 8^C^ | A |  | T | C | G | 9^C^ | T | C | A | A | C | 10^C^ | G | C |
| CH2 | T | T | G | – | G | A | – | T | G | 3^C^ | – | – | – | C | 7^C^ | 8^C^ | A |  | T | A | G | 9^C^ | T | C | A | A | C | 10^C^ | G | C |
| CH3 | T | T | G | – | G | A | 2^C^ | T | G | 3^C^ | – | – | – | C | 7^C^ | 8^C^ | A |  | T | A | G | 9^C^ | T | C | A | A | C | 10^C^ | G | C |
| CH4 | T | T | T | – | G | A | 2^C^ | T | G | 3^C^ | – | – | – | C | 7^C^ | 8^C^ | A |  | T | C | G | 9^C^ | T | C | A | A | C | 10^C^ | G | C |
| CH5 | T | T | T | – | G | A | 2^C^ | T | G | 3^C^ | – | – | – | C | 7^C^ | 8^C^ | A |  | T | C | G | 9^C^ | T | C | G | A | C | 10^C^ | G | C |
| CH6 | T | T | T | – | G | A | – | T | G | 3^C^ | – | – | – | C | 7^C^ | 8^C^ | A |  | T | C | G | 9^C^ | T | C | G | A | C | 10^C^ | G | C |
| CH7 | T | T | T | – | G | A | – | T | G | 3^C^ | – | – | – | C | 7^C^ | 8^C^ | A |  | T | C | G | 9^C^ | G | C | A | A | C | 10^C^ | G | C |
| CH8 | T | T | T | – | G | A | – | T | G | 3^C^ | – | – | – | A | 7^C^ | 8^C^ | A |  | T | C | G | 9^C^ | T | C | A | A | C | 10^C^ | G | C |
| CH9 | T | T | T | – | G | G | – | T | G | 3^C^ | – | – | – | C | 7^C^ | 8^C^ | A |  | T | C | G | 9^C^ | T | C | A | A | C | 10^C^ | G | C |
| CH10 | T | T | T | – | G | A | – | T | G | 3^C^ | – | – | 6^C^ | C | – | 8^C^ | T |  | T | C | G | 9^C^ | T | C | A | A | C | 10^C^ | G | C |
| CH11 | T | T | T | – | G | A | – | T | G | 3^C^ | – | – | – | C | 7^C^ | 8^C^ | A |  | T | C | G | 9^C^ | T | C | A | A | C | 10^C^ | G | A |
| CH12 | T | T | T | – | G | A | – | T | A | 3^C^ | – | – | – | C | 7^C^ | 8^C^ | A |  | T | C | G | 9^C^ | T | C | A | A | C | 10^C^ | G | A |
| CH13 | T | T | T | 1^C^ | G | A | 2^C^ | T | G | 3^C^ | 4^C^ | – | – | C | 7^C^ | 8^C^ | A |  | C | C | G | 9^C^ | T | C | A | C | C | 10^C^ | G | C |
| CH14 | T | T | T | 1^C^ | G | A | – | T | G | 3^C^ | 4^C^ | – | – | C | 7^C^ | 8^C^ | A |  | C | C | G | 9^C^ | T | C | A | C | C | 10^C^ | G | C |
| CH15 | A | A | T | – | G | A | – | T | G | – | 4^C^ | – | – | C | 7^C^ | 8^C^ | A |  | T | C | G | 9^C^ | T | C | A | A | A | – | G | C |
| CH16 | A | A | T | – | T | A | – | A | G | – | 4^C^ | – | – | C | 7^C^ | – | A |  | T | C | G | – | T | C | A | A | A | 10^C^ | G | C |
| CH17 | A | T | T | – | G | A | – | T | G | 3^C^ | 4^C^ | – | – | C | 7^C^ | 8^C^ | A |  | T | C | G | 9^C^ | T | A | A | A | C | 10^C^ | A | C |
| CH18 | A | T | G | – | G | A | – | T | G | 3^C^ | 4^C^ | – | – | C | 7^C^ | 8^C^ | A |  | T | C | A | 9^C^ | T | C | A | A | C | 10^C^ | A | C |
| CH19 | A | T | T | – | G | A | – | T | G | 3^C^ | 4^C^ | 5^C^ | 6^C^ | C | 7^C^ | 8^C^ | A |  | T | C | A | 9^C^ | T | C | A | A | C | 10^C^ | A | C |

Table S3. (continued)

| *Machilus thunbergii* | | | | | | | | | | | | | | |  | *Schima superba* | | | | | | | | | | | | | | |
| --- | --- | --- | --- | --- | --- | --- | --- | --- | --- | --- | --- | --- | --- | --- | --- | --- | --- | --- | --- | --- | --- | --- | --- | --- | --- | --- | --- | --- | --- | --- |
|  | *rpl*32–*trn*L | | | | | | |  | *psb*A–*trn*H | | | | | |  | *rpl*32–*trn*L | | | | | | | | |  | *psb*A–*trn*H | | | | |
|  | 5 | 1 | 2 | 4 | 5 | 6 | 6 |  | 1 |  |  | 1 | 1 | 1 |  |  | 3 |  | 4 | 5 |  | 6 | 8 | 8 |  | 1 | 1 | 1 | 1 | 1 |
| Haplotype | 2 | 3 | 5 | 4 | 0 | 8 | 9 |  | 0 | 1111–1128 | 1303–1307 | 3 | 3 | 3 |  | Haplotype | 2 | 334–338 | 9 | 2 | 604–610 | 1 | 3 | 8 |  | 0 | 2 | 2 | 2 | 3 |
|  |  | 8 | 5 | 8 | 0 | 3 | 0 |  | 8 |  |  | 2 | 5 | 9 |  |  | 3 |  | 3 | 3 |  | 8 | 8 | 5 |  | 1 | 7 | 9 | 9 | 4 |
|  |  |  |  |  |  |  |  |  | 4 |  |  | 5 | 7 | 2 |  |  |  |  |  |  |  |  |  |  |  | 8 | 2 | 3 | 4 | 4 |
| MH1 | A | A | G | T | A | T | G |  | C | – | – | C | G | A |  | SH1 | T | – | T | T | – | C | T | A |  | A | T | T | C | T |
| MH2 | C | C | G | T | A | T | G |  | C | – | – | C | G | A |  | SH2 | T | – | C | T | – | C | T | A |  | A | T | T | C | T |
| MH3 | C | A | G | T | A | T | A |  | C | – | – | C | G | A |  | SH3 | T | – | C | T | – | C | T | C |  | A | T | T | C | T |
| MH4 | C | A | G | T | A | T | G |  | C | – | – | C | G | A |  | SH4 | T | – | C | T | – | C | T | A |  | A | T | G | C | C |
| MH5 | C | A | G | T | A | A | G |  | C | – | – | C | G | A |  | SH5 | T | – | C | T | – | C | T | A |  | A | T | T | C | C |
| MH6 | C | A | G | T | A | A | G |  | T | – | – | C | G | A |  | SH6 | T | - | C | G | 2^S^ | T | A | A |  | G | T | T | C | C |
| MH7 | C | A | G | T | A | A | G |  | C | 1^M^ | – | C | G | A |  | SH7 | A | 1^S^ | C | G | 2^S^ | T | A | A |  | G | T | T | T | C |
| MH8 | C | A | G | C | C | A | G |  | C | – | – | C | A | A |  | SH8 | A | 1^S^ | C | G | 2^S^ | T | A | A |  | G | T | T | C | C |
| MH9 | C | A | G | T | A | A | G |  | C | – | – | T | G | A |  | SH9 | A | 1^S^ | C | G | 2^S^ | T | A | A |  | G | G | T | C | C |
| MH10 | C | A | G | T | A | A | G |  | C | – | – | C | G | C |  |  |  |  |  |  |  |  |  |  |  |  |  |  |  |  |
| MH11 | C | A | A | T | A | A | G |  | C | – | – | C | G | C |  |  |  |  |  |  |  |  |  |  |  |  |  |  |  |  |
| MH12 | C | A | G | T | A | A | G |  | C | – | 2^M^ | C | G | C |  |  |  |  |  |  |  |  |  |  |  |  |  |  |  |  |
| MH13 | A | A | G | T | A | A | G |  | C | – | 2^M^ | C | G | C |  |  |  |  |  |  |  |  |  |  |  |  |  |  |  |  |

Dashes represent indels; 1^C^, TATTATAAGAAATCATAATAA; 2^C^, CGCTTTTATAAATCATTATCAAT; 3^C^, TATTC; 4^C^, CTCTCGTTTATA; 5^C^, TAGTTTAGATAGTTT; 6^C^, TAAAA; 7^C^, AAAAA; 8^C^, TGAAAATA; 9^C^, GACAAAA; 10^C^, ATTATATAA; 1^M^, TTCTTGTTCTATTAAGAG; 2^M^, TTCCT; 1^S^, TATTT; 2^S^, AAAAATG.

**
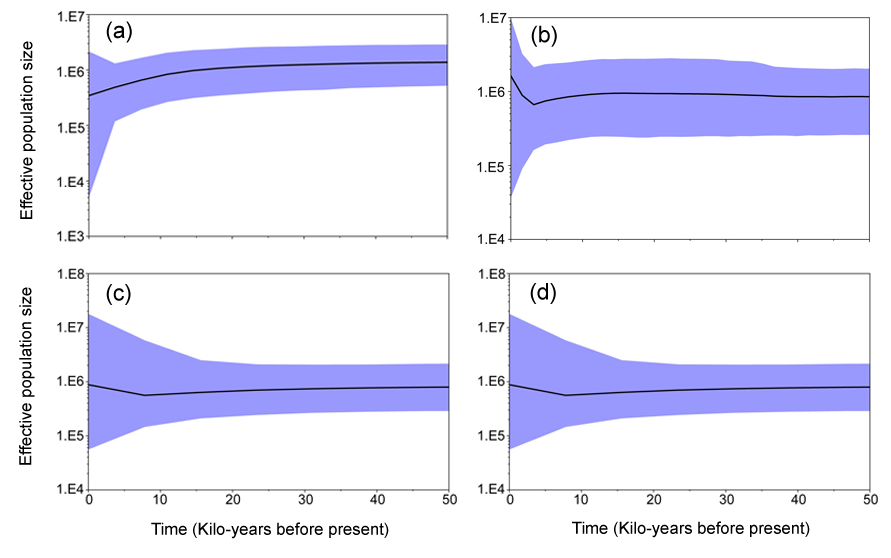
**

Figure S1. Bayesian skyline plot (BSP) inferred from cpDNA data of (a) *Castanopsis tibetana*, (b) *Machilus thunbergii*, (c) western populations of *Schima superba* and (d) eastern populations of *Schima superba*. The black lines are the median posterior of effective population size through time. The blue areas show the limits of 95% highest posterior densities.

**References**

1. Shi, M.M., Michalski, S.G., Welk, E., Chen, X.Y. & Durka, W. Phylogeography of a widespread Asian subtropical tree: genetic east-west differentiation and climate envelope modelling suggest multiple glacial refugia. *J. Biogeogr.* **41**, 1710–1720 (2014).

2. Sun, Y., Hu, H.Q., Huang, H.W. & Vargas-Mendoza, C.F. Chloroplast diversity and population differentiation of *Castanopsis fargesii* (Fagaceae): a dominant tree species in evergreen broad-leaved forest of subtropical China. *Tree Genet. Genomes* **10**, 1531–1539 (2014).

3. Li, J., Ge, X.J., Gao, H.L. & Ye, W.H. Chloroplast DNA diversity in *Castanopsis hystrix* populations in south China. *Forest Ecol. Manag.* **243**, 94–101 (2007).

4. Gong, W. *et al*. From glacial refugia to wide distribution range: demographic expansion of *Loropetalum chinense* (Hamamelidaceae) in Chinese subtropical evergreen broadleaved forest. *Org. Divers. Evol.* **16**, 23–38 (2016).

5. Xu, J. *et al*. Phylogeography of *Quercus glauca* (Fagaceae), a dominant tree of East Asian subtropical evergreen forests, based on three chloroplast DNA interspace sequences. *Tree Genet. Genomes* **11**, 805 (2015).

6. Qi, X.S. *et al.* Molecular data and ecological niche modelling reveal a highly dynamic evolutionary history of the East Asian Tertiary relict *Cercidiphyllum* (Cercidiphyllaceae). *New Phytol.* **196**, 617–630 (2012).

7. Kou, Y.X. *et al*. The antiquity of *Cyclocarya paliurus* (Juglandaceae) provides new insights into the evolution of relict plants in subtropical China since the late Early Miocene. *J. Biogeogr.* **43**, 351–360 (2016).

8. Zhang, Y.H., Wang, L.J., Comes, H.P., Peng, H. & Qiu, Y.X. Contributions of historical and contemporary geographic and environmental factors to phylogeographic structure in a Tertiary relict species, *Emmenopterys henyi* (Rubiaceae). *Sci. Rep.* **6**, 24041 (2016).

9. Gao, Y.N., Comes, H.P., Sakaguchi, S., Chen, L.Y. & Qiu, Y.X. Evolution of East Asia’s Arcto-Tertiary relict *Euptelea* (Eupteleaceae) shaped by Late Neogene vicariance and Quaternary climate change. *BMC Evol. Biol.* **16**, 66 (2016).

10. Wang, J., Gao, P.X., Kang, M., Lowe, A.J. & Huang, H.W. Refugia within refugia: the case study of a canopy tree *Eurycorymbus cavaleriei* in subtropical China. *J. Biogeogr.* **36**, 2156–2164 (2009).

11. Liu, M.H. Phylogeography of *Fagus longipetiolata*: insights from nuclear DNA microsatellites and chloroplast DNA variation. (East China Normal University, Shanghai, PhD dissertation, 2008).

12. Li, X.H., Shao, J.W., Lu, C., Zhang, X.P. & Qiu, Y.X. Chloroplast phylogeography of a temperate tree *Pteroceltis tatarinowii* (Ulmaceae) in China. *J. Syst. Evol.* **50**, 325–333 (2012).

13. Sun, Y. *et al*. Chloroplast phylogeography of the East Asian Arcto-Tertiary relict *Tetracentron sinense* (Trochodendraceae). *J. Biogeogr.* **41**, 1721–1732 (2014).

14. Chen, D. *et al*. Phylogeography of *Quercus variabilis* based on chloroplast DNA sequence in East Asia: multiple glacial refugia and Mainland-migrated island populations. *PloS one* **7**, e47268 (2012).

15. Li, Y., Yan, H.F. & Ge, X.J. Phylogeographic analysis and environmental niche modelling of widespread shrub *Rhododendron simsii* in China reveals multiple glacial refugia during the last glacial maximum. *J. Syst. Evol.* **50**, 362–373 (2012).

16. Wang, H.W. & Ge, S. Phylogeography of the endangered *Cathaya argyrophylla* (Pinaceae) inferred from sequence variation of mitochondrial and nuclear DNA. *Mol. Ecol.* **15**, 4109–4122 (2006).

17. Tian, S., Lopez-Pujol, J., Wang, H.W., Ge, S. & Zhang, Z.Y. Molecular evidence for glacial expansion and interglacial retreat during the Quaternary climatic changes in a montane temperate pine (*Pinus kwangtungensis* Chun ex Tsiang) in southern China. *Plant Syst. Evol.* **284**, 219–229 (2010).

18. Gao, L.M. *et al*. High variation and strong phylogeographic pattern among cpDNA haplotypes in *Taxus wallichiana* (Taxaceae) in China and North Vietnam. *Mol. Ecol.* **16**, 4684–4698 (2007).

19. Tian, S. *et al*. Repeated range expansions and inter-/postglacial recolonization routes of *Sargentodoxa cuneata* (Oliv.) Rehd. et Wils. (Lardizabalaceae) in subtropical China revealed by chloroplast phylogeography. *Mol. Phylogenet. Evol.* **85**, 238–246 (2015).

20. Wang, Y.H. *et al*. Molecular phylogeography and ecological niche modelling of a widespread herbaceous climber, *Tetrastigma hemsleyanum* (Vitaceae): insights into Plio-Pleistocene range dynamics of evergreen forest in subtropical China. *New Phytol.* **206**, 852–867 (2015).
